# miR-29a-3p/*Vegfa* axis modulates high phosphate-induced vascular smooth muscle cell calcification

**DOI:** 10.1080/0886022X.2025.2489712

**Published:** 2025-04-22

**Authors:** Chen Fu, Qiaojing Liang, Lili Ma, Wenhu Liu, Weikang Guo, Gang Wang

**Affiliations:** a Department of Nephrology, National Center for Orthopaedics, Beijing Jishuitan Hospital, Capital Medical University, Beijing, China; b Department of Nephrology, Faculty of Kidney Diseases, Beijing Tongren Hospital, Capital Medical University, Beijing, China; c International Medical Center, Beijing Friendship Hospital, Capital Medical University, Beijing, China; d Department of Nephrology, Faculty of Kidney Diseases, Beijing Friendship Hospital, Capital Medical University, Beijing, China

**Keywords:** miR-29a, vascular calcification, vascular smooth muscle cells, *Vegfa*, chronic kidney disease

## Abstract

Vascular calcification (VC) is a major contributor to the progression of cardiovascular disease (CVD). The VC is characterized by arterial stiffness and impaired blood flow. This pathology is especially prevalent in chronic kidney disease (CKD), where dysregulated mineral metabolism and elevated phosphate levels accelerate calcification of vascular smooth muscle cells (VSMCs). Emerging evidence suggests that microRNAs (miRNAs) are key regulators of VC, with the miR-29 family implicated in extracellular matrix remodeling and calcification. We investigated the role of the miR-29a-3p/vascular endothelial growth factor A (*Vegfa*) axis in CKD-associated VC. Dual-luciferase assays and bioinformatic analysis confirmed that miR-29a-3p directly targets *Vegfa*, a critical regulator of vascular homeostasis. miR-29a-3p overexpression significantly attenuated VSMC calcification under high phosphate conditions, as indicated by significantly reduced Alizarin Red staining (ARS, *P* < 0.0001) and intracellular calcium content (ICC, *P* = 0.0235). Conversely, *Vegfa* overexpression exacerbated calcification (*P* = 0.0010 for ICC and *P* = 0.0001 for ARS). *Vegfa* knockdown mitigated these effects (*P* < 0.0001 for both ARS and ICC). Notably, miR-29a-3p counteracted calcification even in *Vegfa*-overexpressing cells (*P* < 0.0001 for ARS and *P* = 0.0235 for ICC), underscoring its protective role in vascular integrity. These findings highlight the therapeutic potential of targeting the miR-29a-3p/*Vegfa* axis for VC management in patients with CKD. miRNA-based interventions may offer a promising strategy for preventing pathological calcification and reducing the risk of CVD in affected patients.

## Introduction

Vascular calcification (VC) is the pathological deposition of hydroxyapatite (HA) as bioapatite in the vascular system. VC contributes to the development of cardiovascular diseases (CVDs) by affecting the ability of vascular smooth muscle cells (VSMCs) to regulate luminal diameter [1], leading to arterial stiffness, elevated blood pressure, and decreased blood flow [2,3]. Chronic kidney disease (CKD) is a progressive condition in which the gradual loss of renal function leads to CVDs and exacerbates VC by disrupting mineral metabolism [4] and increasing phosphate levels [5]. Both exacerbations contribute to the calcification process of VSMCs [2,6]. VC frequently coexists with CKD and has been linked to higher morbidity and mortality [7–9].

Recent studies have identified microRNAs (miRNAs) as diagnostic and prognostic markers of diseases associated with VC [10,11]. These small non-coding RNAs bind to messenger RNA (mRNA) 3′-untranslated region (UTR) sequences, reducing mRNA stability and translation into proteins [12]. miRNAs also influence VC by modulating the phenotypes of VSMCs [13,14], regulating calcium phosphate metabolism [15,16], and affecting inflammatory responses within the vascular system [17,18]. For instance, miR-29a, miR-29b, and miR-29c, which are all members of the miR-29 family, share a conserved seed sequence, target similarly predicted genes, and regulate extracellular matrix-related genes across various cell types, including VSMCs [19,20]. The expression of miR-29a/b was reportedly reduced in the calcified arteries of nephrectomized rats treated with calcitriol and in the radial arteries of patients with CKD, while its inhibition enhanced VC through the upregulation of A disintegrin and metalloproteinase-7 (ADAMTS7), a metalloproteinase associated with the risk of CVD risk [21]. Furthermore, miR-29a-3p inhibition can suppress VSMC proliferation, migration, and phenotypic switching to a synthetic state, thereby mitigating the progression of aortic dissection in rats [22]. Similarly, in models of atherosclerosis (AS) induced by oxidized low-density lipoprotein, miR-29a-3p overexpression can reduce the proliferation, migration, and invasion of VSMCs [23]. In addition, miR-29a-3p helps protect against AS by reducing plaque formation and arterial wall thickening, as demonstrated in ApoE−/− mice [24]. Overexpression of miR-29a-3p reduces the aortic plaque area, and this reduction is linked to increased plaque formation. The collective findings have implicated miR-29a-3p as a pivotal regulator linking VSMC function, VC, and inflammatory modulation, highlighting its potential as a therapeutic target in the intertwined pathology of AS and CKD.

An increasing body of recent research has established the tumor suppressor properties of the miR-29 family through the identification and targeting of vascular endothelial growth factor (*Vegfa*), which is essential for the survival of vascular endothelial cells and formation of blood vessels [25–27]. In particular, miR-29a-3p regulates *Vegfa* expression by directly targeting its 3′-UTR, suppressing *Vegfa* levels at both mRNA and protein levels [28–30]. This regulation influences angiogenesis [29,31,32], tumor progression [28,30,33,34], and fibrosis [35–37]. Overexpression of miR-29a-3p reduces *Vegfa* [28–30,34] and inhibits angiogenesis and cell proliferation, whereas its inhibition increases *Vegfa* expression [21,28]. The miR-29a-3p/*Vegfa* axis plays a distinct role in various diseases. In preeclampsia, reduced *Vegfa* levels disrupt placental vascular development; miR-29a-3p contributes to this disruption by directly targeting *Vegfa* mRNA, leading to its downregulation and impaired angiogenesis [29]. In atrial fibrillation, low miR-29a-3p levels increase fibroblast activity by increasing *Vegfa* expression, which promotes cardiac fibroblast proliferation and collagen release [36]. In cancers, including hepatocellular and gastric, miR-29a-3p acts as a tumor suppressor by downregulating *Vegfa*, reducing metastasis and improving outcomes [25,28,30,38]. It also modulates oxidative stress responses in glaucoma and angiogenesis during myocardial infarction [31,32]. Reduced miR-29a-3p expression and upregulated *Vegfa* levels contribute to cardiac fibrosis, with miR-29a-3p acting as a suppressor by directly targeting and downregulating *Vegfa* [39]. Furthermore, *Vegfa* polymorphisms such as rs699947 have been implicated in VC and end-stage renal disease (ESRD) susceptibility [40,41], although their protective or detrimental roles remain to be investigated. Elevated *Vegfa* levels, which correlate with CKD severity and declining renal function, are also linked to renal fibrosis and inflammation through pathways like nuclear factor-kappa B and transforming growth factor-β signaling [42–44]. *Vegfa* further interacts with hypoxia-induced and endoplasmic reticulum stress pathways, contributing to vascular rarefaction and fibrosis [44]. Finally, *Vegfa* is the critical secreted factor that, in cooperation with bone morphogenetic protein-4 (*Bmp-4*) and *Wnt3a*, induces the ectopic osteoblastic transformation and calcification of VSMCs [45–47]. The intricate roles of *Vegfa* in cardiomyopathy, CKD and ESRD underscores its significance as a biomarker [48] and potential therapeutic target [44,49].

The association between miR-29a-3p and *Vegfa*, as well as the impact of *Vegfa* on the calcification of VSMCs, offers novel insights into the molecular mechanisms underlying VC in CKD and suggests potential treatment targets to inhibit this process. In this study, we investigated the link between miR-29a-3p and *Vegfa*, the well-documented member of the *Vegf* family, and analyzed the functional impact of this interaction on VC, particularly under high phosphate conditions associated with CKD.

## Results

### Direct targeting of *Vegfa* by miR-29a-3p in VSMCs

With increasing evidence supporting the role of miR-29a-3p in VSMC calcification [21,22,24], we aimed to identify the miR-29a-3p targets in these cells. Various miRNA target prediction tools utilize distinct algorithms based on sequence complementarity, evolutionary conservation, and thermodynamic stability of miRNA:mRNA duplexes [50]. However, these tools can produce false positives or negatives due to methodological differences. To enhance the prediction accuracy and minimize bias, we employed a stringent approach, selecting only targets identified by the intersection of four well-established tools—miRDB [51], miRTarBase [52], miRWalk [53], and TargetScan [54]. These tools complement each other; miRTarBase prioritizes experimentally validated targets, TargetScan incorporates evolutionary conservation, and miRDB and miRWalk apply advanced predictive algorithms.

**Figure 1.**
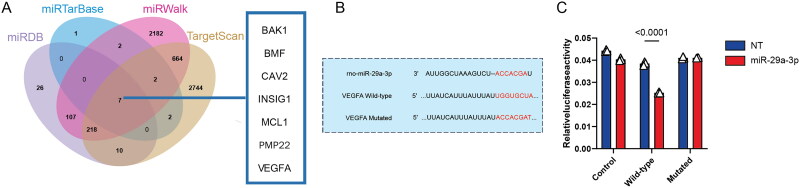
Identification of *Vegfa* as a direct target of miR-29a-3p in VSMCs using bioinformatics and dual-luciferase assay. (A) Venn diagram depicting the overlap of miR-29a-3p target genes predicted by four miRNA target prediction tools: miRTarBase, miRWalk, TargetScan, and miRDB. The box highlights the seven genes identified as common targets across all tools. (B) Sequence alignment of miR-29a-3p with the 3′-UTR region of wild-type and mutated *Vegfa* mRNA. The predicted binding site and seed region are marked in red. (C) Dual-luciferase reporter assay results for VSMCs co-transfected with a control vector, a vector containing the wild type (WT) *Vegfa* 3′-UTR sequence, or the MUT *Vegfa* sequence, along with miR-29a-3p mimics or a negative control miRNA (NT). Relative luciferase activity was measured, and data are presented as mean ± SD from three independent experiments.

By integrating the predictions from these databases, we identified seven commonly predicted miR-29a-3p targets: *Bak1, Bmf, Cav2, Insig1, Mcl1, Pmp22*, and *Vegfa* (Figure 1(A)). Given the emerging role of *Vegfa* in VC [55], we validated its interaction with miR-29a-3p. We cloned rat *Vegfa* 3′-UTR, which contains the predicted miR-29a-3p binding site (Figure 1(B)), into the psiCHECK-2 vector and performed a dual-luciferase reporter assay (Figure 1(C)) in VSMCs, using a mutated 3′-UTR as a negative control. Transfection of the *Vegfa* 3′-UTR reporter construct, alongside the miR-29a-3p mimic, resulted in a significant decrease in luciferase activity compared to a non-targeting (NT) miRNA control (0.025 ± 0.0004 vs. 0.039 ± 0.0009 AU, respectively; *P* < 0.0001), confirming a direct interaction (Figure 1(C)). In contrast, co-transfection with the mutated *Vegfa* 3′-UTR showed no significant reduction in luminescence, reinforcing that miR-29a-3p directly regulates *Vegfa* post-transcriptionally.

### miR-29a-3p modulates *Vegfa* expression under high phosphate conditions

**Figure 2.**
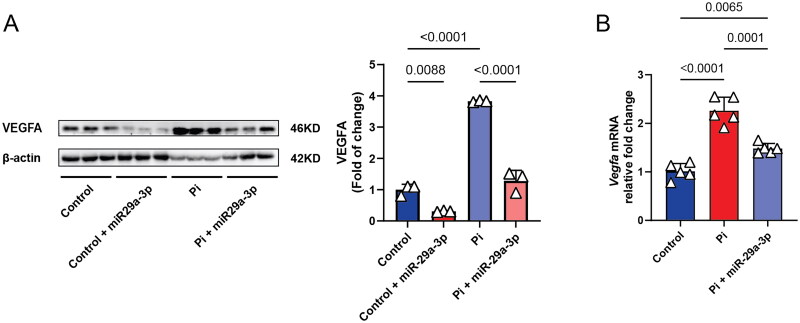
miR-29a-3p overexpression suppresses *Vegfa* expression in VSMCs. (A) Representative Western blot analysis of VEGFA protein levels in VSMCs under standard culture conditions (control), high phosphate conditions (Pi), and high phosphate conditions following miR-29a-3p overexpression (Pi + miR-29a-3p). β-actin served as a loading control. Quantitative analysis of VEGFA protein levels is shown as relative fold change normalized to β-actin. (B) *Vegfa* mRNA levels quantified using RT-qPCR in the same experimental groups. GAPDH was used as an internal control for normalization. Data represent the mean ± SD of five independent biological replicates.

High phosphate levels drive VSMC calcification [56,57]. As shown in Supplemental Fig. 1, miR-29a-3p expression was significantly upregulated in VSMCs cultured in high phosphate media compared to controls (9.656 ± 7.020 vs. 2.393 ± 1.870 AU; *P* = 0.0356), suggesting a role in the calcification process. To explore whether miR-29a-3p regulates endogenous *Vegfa* expression under these conditions, we examined VEGFA protein and mRNA levels in VSMCs treated with high phosphate. Western blot analysis (Figure 2(A)) revealed a significant increase in VEGFA protein levels compared to controls (3.831 ± 0.02798 vs. 1.000 ± 0.1726 AU; *P* < 0.0001), while miR-29a-3p overexpression reduced VEGFA levels to near-normal (1.284 ± 0.3331 AU; *P* < 0.0001). Similarly, quantitative real-time polymerase chain reaction (RT-qPCR; Figure 2(B)) confirmed that miR-29a-3p overexpression significantly downregulated *Vegfa* mRNA levels relative to the NT control (1.482 ± 0.1043 vs. 2.260 ± 0.2784 AU; *P* < 0.0001). These findings suggest that *Vegfa* upregulation contributes to phosphate-induced VSMC calcification, and that miR-29a-3p may counteract this effect by suppressing *Vegfa* expression.

### *Vegfa* knockdown attenuates high phosphate-induced VSMC calcification

**Figure 3.**
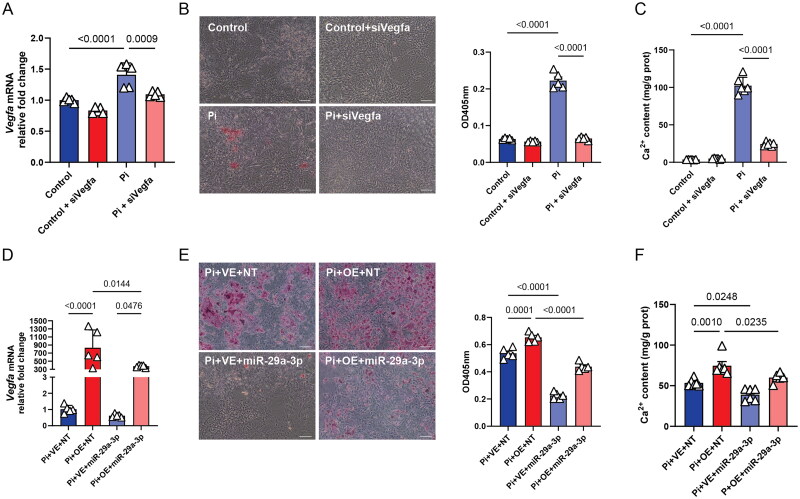
*Vegfa* knockdown and miR-29a-3p overexpression mitigate high phosphate-induced VSMCs calcification. (A–C) Analysis of *Vegfa* knockdown effects in VSMCs cultured in normal media (control) or high phosphate conditions (Pi). (A) *Vegfa* mRNA levels were quantified via RT-qPCR. (B) ARS with corresponding spectrophotometric quantification to assess cellular mineralization. (C) Quantification of intracellular Ca^2+^ content. Cells were treated with small interfering RNA targeting *Vegfa* (siVegfa) or a NT control. (D–F) Investigation of the role of miR-29a-3p in VSMCs cultured under high phosphate conditions. (D) *Vegfa* mRNA expression measured using RT-qPCR, (E) mineralization assessed using ARS and spectrophotometric quantification, and (F) intracellular Ca^2+^ levels were quantified. Cells were transfected with either an empty vector (VE) or a *Vegfa*-overexpressing construct (OE), combined with a NT miRNA mimic or a miR-29a-3p mimic. All experiments were performed in five replicates, and error bars represent the SD. GAPDH was used as an internal control in RT-qPCR experiments. Scale bar = 100 µm.

Given the observed increase in VEGFA protein levels under high phosphate culture conditions, we investigated the role of endogenously expressed *Vegfa* in VSMC calcification. Transfection of siRNA targeting *Vegfa* into VSMCs cultured in standard medium had a negligible effect on *Vegfa* levels compared to controls (0.8340 ± 0.04827 vs. 1.000 ± 0.03742 AU, *P* = 0.0846). However, under high phosphate conditions, *Vegfa* knockdown successfully restored *Vegfa* levels to near-normal (1.096 ± 0.04615 vs. 1.410 ± 0.1884 AU, *P* = 0.0009), as confirmed by RT-qPCR analysis (Figure 3(A)).

Furthermore, *Vegfa* knockdown significantly reduced VSMC calcification, as demonstrated by Alizarin Red staining [ARS, optical density at 450 nm (OD450) of 0.05673 ± 0.002006 vs. 0.2226 ± 0.02216; *P* < 0.0001; Figure 3(B)] and intracellular calcium quantification (24.47 ± 2.696 vs. 102.3 ± 11.12 mg/g total protein, *P* < 0.0001; Figure 3(C)). These findings suggest that *Vegfa* plays a pivotal role in the pathological calcification of VSMCs, highlighting its potential as a therapeutic target for preventing VC.

### miR-29a-3p overexpression mitigates VSMC calcification under high phosphate conditions

To further investigate the roles of *Vegfa* and miR-29a-3p in VSMC calcification, we assessed the effects of their overexpression in VSMCs cultured under high phosphate conditions. Transfection with a *Vegfa*-expressing plasmid significantly elevated *Vegfa* mRNA levels (833.4 ± 440.9 vs. 1.021 ± 0.2374 AU; *P* < 0.0001; Figure 3(D)). However, co-expression of miR-29a-3p markedly reduced *Vegfa* mRNA levels (376.5 ± 30.86 AU; *P* = 0.0476), confirming that miR-29a-3p can suppress constitutive *Vegfa* overexpression.

*Vegfa* overexpression exacerbated high phosphate-induced VSMC calcification, as evidenced by increased ARS (OD450 of 0.6536 ± 0.03288 vs. 0.5362 ± 0.03733; *P* = 0.0001; Figure 3(E)) and elevated intracellular calcium levels (74.61 ± 12.33 vs. 53.46 ± 4.489 mg/g total protein; *P* = 0.0010; Figure 3(F)). Conversely, miR-29a-3p overexpression significantly attenuated VSMC calcification under high phosphate conditions, yielding reductions in both ARS (OD450 of 0.4385 ± 0.02920; *P* < 0.0001; Figure 3(E)) and intracellular calcium content (ICC, 60.02 ± 5.386 mg/g total protein; *P* = 0.0235; Figure 3(F)), similar to the effect observed with *Vegfa* knockdown.

Additionally, miR-29a-3p substantially reduced calcification in cells expressing endogenous *Vegfa*, as demonstrated by lower ARS (OD450 of 0.2252 ± 0.02407; *P* < 0.0001; Figure 3(E)) and decreased intracellular calcium levels (38.99 ± 7.412 mg/g total protein; *P* = 0.0248; Figure 3(F)). These findings suggest that miR-29a-3p plays a protective role against phosphate-induced VSMC calcification by modulating *Vegfa* expression.

## Discussion

Pathological mineral deposition in the vascular system remains a significant predictor of CVD morbidity and mortality in patients with CKD. An essential aspect of studying VC in CKD is the interplay between miRNAs and *Vegfa*, a gene crucial for vascular endothelial function and angiogenesis [25–27]. This study focused on the interaction between miR-29a-3p and *Vegfa* and revealed a potential therapeutic mechanism for reducing VC in CKD. Our key findings highlight the regulatory role of miR-29a-3p in *Vegfa* expression and subsequent calcification of VSMCs under high phosphate conditions, which are the hallmarks of CKD.

One of the primary findings of this study is that miR-29a-3p directly targets *Vegfa* in VSMCs under high phosphate conditions. This result is consistent with previous studies showing that miR-29a, particularly the miR-29/*Vegfa* axis, acts as a key regulator of vascular homeostasis [25,28,39]. Previous studies have documented that miR-29a-3p influences extracellular matrix composition [14,35–37] and VC by targeting specific genes involved in calcification pathways [21]. Our findings expand this knowledge by providing a mechanistic explanation for the regulatory role of the miR-29/*Vegfa* axis in CKD-associated VC, further corroborating its involvement in vascular pathophysiology.

The observation that increased miR-29a-3p expression inhibits VSMCs calcification underlines the protective role of this miRNA. Similar protective effects have been reported in studies where miR-29a-3p mitigated vascular remodeling and plaque formation in AS models [23,24]. The inhibition of VSMCs calcification by miR-29a-3p overexpression suggests that this miRNA not only regulates gene targets, such as *Vegfa*, but also preserves the structural integrity of the vasculature by maintaining extracellular matrix stability. This reinforces the potential of miR-29a-3p as a therapeutic target, as its modulation may prevent or reverse VC progression in patients with CKD [21].

Another pivotal finding was that decreasing *Vegfa* expression attenuated calcification in VSMCs. *Vegfa* has been widely studied for its angiogenic properties [25–27]. However, its role in calcification remains poorly explored [40–44]. Our findings suggest that *Vegfa* contributes to calcification under pathological conditions, possibly by promoting vascular inflammation or dysregulating mineral metabolism. This suggestion aligns with evidence from studies on other vascular diseases, where *Vegfa* was shown to play a dual role, being beneficial under normal conditions but potentially harmful when dysregulated. By demonstrating that *Vegfa* reduction prevents calcification, this study highlights a novel therapeutic avenue for targeting *Vegfa* in VC management.

These findings have profound implications, particularly in the context of CKD, where VC contributes significantly to cardiovascular morbidity and mortality. The miR-29a-3p/*Vegfa* axis represents a promising target for therapeutic interventions aimed at preserving vascular health. Modulating this axis could offer a dual benefit: mitigating VC and addressing associated complications, such as arterial stiffness and impaired blood flow. These findings advance our understanding of miRNA-mediated regulatory mechanisms in CKD and provide a framework for the development of miRNA-based therapies.

This study has several limitations. Firstly, the findings were primarily derived from *in vitro* models, which may not fully replicate the complexity of *in vivo* conditions. Although these models provide valuable mechanistic insights, further validation using animal models and clinical studies is necessary to confirm their translatability. Secondly, this study focused solely on the miR-29a-3p/*Vegfa* axis, leaving the potential interactions of miR-29a-3p with other regulatory genes involved in calcification unexplored. In addition, the long-term effects and safety of targeting this axis in a therapeutic context need to be investigated.

In conclusion, this study provides compelling evidence that the miR-29a-3p/*Vegfa* axis plays a critical role in modulating VC in patients with CKD. By elucidating the molecular mechanisms underlying this interaction, we offer new insights into the pathogenesis of VC and identify potential therapeutic targets. Future studies should aim to validate these findings *in vivo*, explore the broader regulatory network of miR-29a, and assess the clinical feasibility of targeting this axis. These efforts will be crucial for translating these discoveries into effective treatments to improve the vascular health of patients with CKD.

## Materials and methods

### Bioinformatics analysis for miRNA target prediction

To identify the putative targets of miR-29a-3p, we used a combination of four widely recognized miRNA target prediction tools: miRDB, miRTarBase, miRWalk, and TargetScan. These tools were selected because of their complementary strengths and widespread acceptance in the field of miRNA research, ensuring a comprehensive and reliable analysis. miRTarBase was included because it provides a curated database of experimentally validated miRNA target interactions, making it particularly useful for identifying high-confidence targets. TargetScan was chosen because of its ability to identify evolutionarily conserved miRNA-binding sites across species, which enhanced the biological relevance of the predictions. miRDB utilizes a machine learning-based algorithm trained on experimental data to predict functional targets. miRWalk includes an extensive database of potential binding sites throughout the gene sequence, not limited to the 3′-UTR, providing additional predictive depth. To improve the reliability of our predictions and minimize false positives, we applied stringent selection criteria by considering only targets identified by all four tools. This consensus approach leverages the unique strengths of each tool, ensuring higher confidence in the predicted targets and reducing the impact of individual algorithm bias.

These tools analyze key parameters such as the complementarity between the miRNA seed region and the 3′-UTR of target mRNAs, thermodynamic stability of the miRNA:mRNA duplex, and evolutionary conservation. These features confirm that the identified targets are biologically plausible and functionally relevant. Additionally, we used the default parameters provided by each tool to maintain the reproducibility and transparency of our analysis. The predicted targets were validated experimentally using a dual-luciferase reporter assay, which confirmed a direct interaction between miR-29a-3p and its targets.

### Dual-luciferase reporter gene assay

A dual-luciferase reporter assay was performed for experimental validation. The 3′-UTR of the rat *Vegfa* gene, containing the predicted miR-29a-3p binding site, was cloned into the psiCHECK-2 vector (Promega, USA; Cat. #C8021). A negative control plasmid was constructed by mutagenizing the putative miR-29a-3p binding site using QuickMutation^™^ Site-directed Gene Mutagenesis Kit (Beyotime Co., China; Cat. #0206S). VSMCs cultured in 96-well plates were co-transfected with 100 ng/well plasmid DNA and 100 nM/well of miR-29a-3p mimics (RiboBio Co., China; Cat. #miR10000802-1-5) using Lipofectamine 3000 (Thermo Fisher Scientific, USA; Cat. #L3000015). Luciferase activity was measured after 48 h to assess the direct interaction between miR-29a-3p and the *Vegfa* 3′-UTR, with a significant decrease in activity indicating successful targeting.

### Culture and treatments of VSMCs

Primary VSMCs from Sprague-Dawley rats (Cell Biologics, USA; Cat. #RA-6080) was routinely cultured in complete medium containing Dulbecco’s modified Eagle’s medium (DMEM) (Gibco, USA; Cat. #C11965500BT) with 4500 mg/L. The medium was supplemented with 10% fetal bovine serum FBS (Gibco, Australia; Cat. #10099141 C) with 100 U/mL penicillin and 100 µg/mL streptomycin. VSMCs collected after three to five passages were used for *in vitro* studies. The cells were cultured at 37 °C in a humid environment of 5% CO_2_. The medium was changed every two days. For high phosphate treatment, the cells were maintained in DMEM supplemented with 4.0 mM Na_2_HPO_4_/NaH_2_PO_4_ for 12 days. *Vegfa* and miR-29a-3p overexpression were performed in 12-well plates by transfecting 1 μg of plasmid DNA (RiboBio Co., China; Cat. #GEP00266) or 200 nM miRNA mimics (RiboBio Co., China; Cat. #miR10000802-1-5) and 2 µL/well of Lipofectamine 3000 (Thermo Fisher Scientific, USA; Cat. #L3000015) according to the manufacturer’s instructions. *Vegfa* knockdown was performed by transfecting cells with 200 nM *Vegfa* siRNA (RiboBio Co., China; Cat. #siBDM2500) and 2 µL/well of Lipofectamine. Subsequent experiments were performed 48 h after transfection.

### Western blot analysis

VSMCs recovered by centrifugation were homogenized and separated using 10% sodium dodecyl sulfate-polyacrylamide gel electrophoresis (China National Pharmaceutical Group Corporation Chemical Reagent Co., Ltd., China; Cat. #30166428). The proteins were then transferred to polyvinylidene fluoride membranes (Millipore Co., USA; Cat. #ISEQ00010). The blots were stained with rabbit polyclonal antibodies against VEGFA (1:10,000; Proteintech, USA; Cat. #19003-1-AP), and mouse monoclonal antibodies against β-Actin (1:10,000, Servicebio, China; Cat. #GB15001), followed by anti-rabbit IgG (1:10,000, SIMUBIOTECH, China; Cat. #S2001), and anti-mouse IgG horseradish peroxidase conjugates (1:10,000, ABclonal, China; Cat. #AS003). For visualization, the membranes were soaked for 1 min in the ECL reagent (Applygen, China; Cat. #P1050), and imaging was done using Chemiluminescent imaging system (Sinsage Co., China; Cat. #MiniChemi 610).

### RNA extraction and mRNA quantification via RT-qPCR

Total RNA was extracted from frozen 106 VSMCs pellets using an RNeasy Kit (Qiagen, USA; Cat. #74104), according to the manufacturer’s instructions. The TaqMan RNA-to-CT 1-Step Kit (Thermo Fisher Scientific, USA; Cat. #4392653) was used for reverse transcription and real-time quantitative PCR with 5 ng RNA per well. Each reaction was performed in a technical replicate on the QuantStudio^™^ 5 Real-Time PCR System (Thermo Fisher Scientific, USA, Cat. #A34322). The following commercial TaqMan primers were used: rat *Vegfa* primer (Beijing Tsingke Biotech Co., Ltd., China) and rat glyceraldehyde 3-phosphate dehydrogenase (GAPDH; Beijing Tsingke Biotech Co., Ltd., China). The 2^−ΔΔCt^ technique [58] was employed to calculate relative RNA expression, with GAPDH serving as an internal control.

### ARS and quantification of calcium

To evaluate the calcification of VSMCs, ARS was performed as previously described [59]. In brief, following treatment under different conditions, VSMCs cultured on 12 well plates were fixed in 4% paraformaldehyde in PBS for 15 min at 20 °C. The samples were rinsed with distilled water and stained with ARS solution (0.2%, pH 8.3; Solarbio, China; Cat. #G1450) for 20 min. They were then rinsed again with distilled water and examined using a model DM3000 microscope (, Germany). Alizarin Red stained cells were lysed with 10% (v/v) acetic acid (Merck, Germany), Leica and mineralization was quantified spectrophotometrically. Aliquots of the supernatant were read in triplicate at 405 nm in a 96-well plate using opaque-walled, transparent-bottomed plates (Fisher Life Sciences, USA), as previously described [60]. The ICC was directly quantified using a colorimetric Calcium Assay Kit (Sigma-Aldrich, USA; Cat. #MAK022).

### Statistical analyses

All experiments were performed using at least three biological replicates, unless otherwise stated. Data are expressed as mean ± standard deviation (SD). Statistical significance was assessed using GraphPad Prism 9.0 (GraphPad Software, USA). Comparisons between two groups were performed using a paired two-tailed Student’s *t*-test, whereas one-way analysis of variance followed by Tukey’s *post hoc* test was used for comparisons among multiple groups. Normality was tested using the Shapiro-Wilk test, and homogeneity of variance was assessed using Levene’s test. If the data did not meet parametric assumptions, the Kruskal-Wallis test followed by Dunn’s multiple comparison test was applied.

## Supplementary Material

Uncropped western blots .docx

Supp Fig 1.png

## Data Availability

Data supporting the findings of this study are available from the corresponding author upon request.
