# Supplementary material for: miR-29a-3p/Vegfa axis modulates high phosphate-induced vascular smooth muscle cell calcification
Source: Ren Fail. 2025 Apr 22;47(1):2489712. doi: 10.1080/0886022X.2025.2489712 (PMC12016250; doi:10.1080/0886022X.2025.2489712)

Sample sequence left to right: 1) Control，2) Pi，3) Pi+miR-29, 4) Control，5) Pi，6) Pi+miR-29; 7) Control，8) Pi，9) Pi+miR-29. Molecular Ladder visible on the far left.


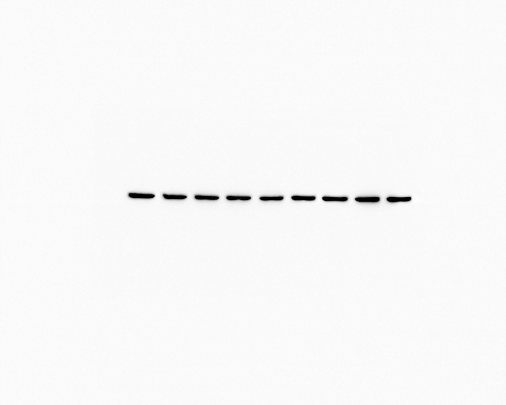
uncropped β-actin

cropped β-actin


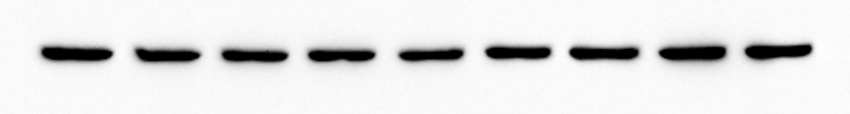


membrane β-actin


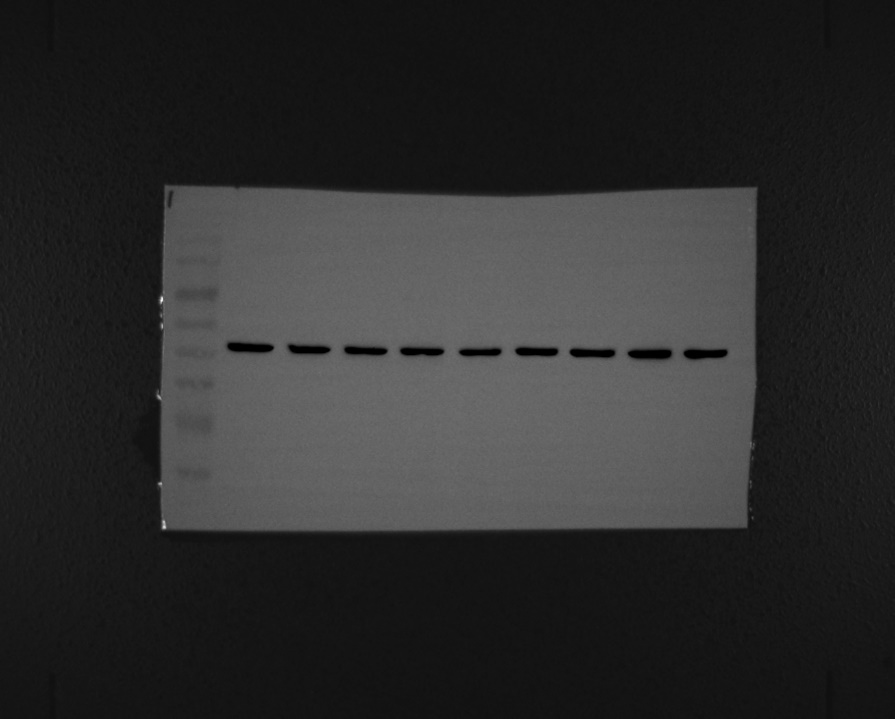


Uncropped VEGFA


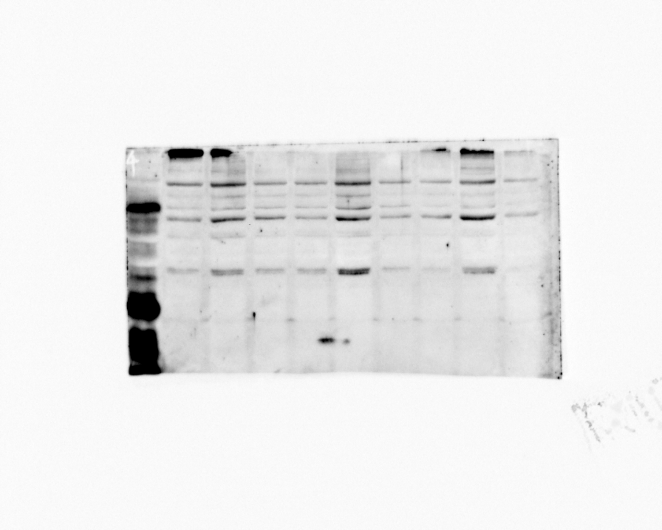


Cropped VEGFA


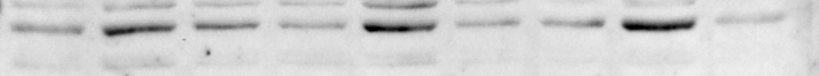


Membrane VEGFA


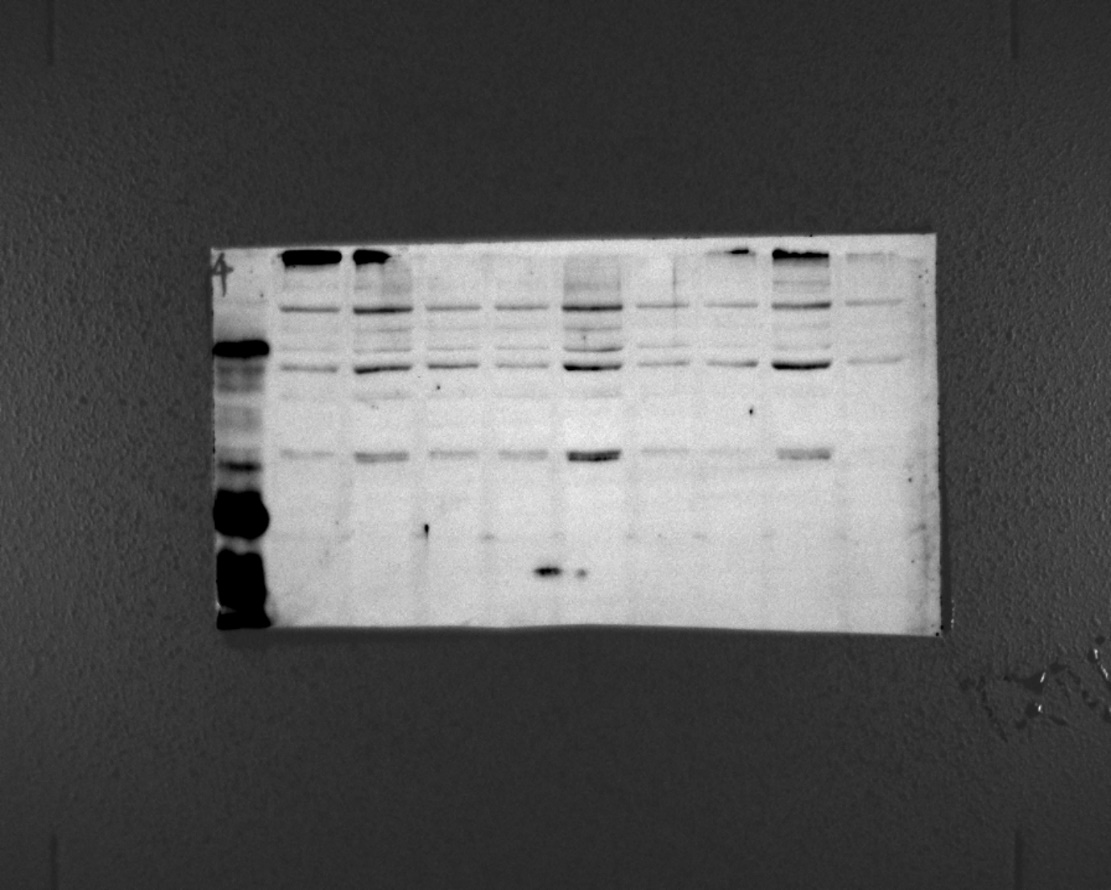

Supplement: Uncropped western blots .docx [file IRNF_A_2489712_SM6943.docx]
