# Supplementary figures and images for: miR-29a-3p/Vegfa axis modulates high phosphate-induced vascular smooth muscle cell calcification
Source: Ren Fail. 2025 Apr 22;47(1):2489712. doi: 10.1080/0886022X.2025.2489712 (PMC12016250; doi:10.1080/0886022X.2025.2489712)

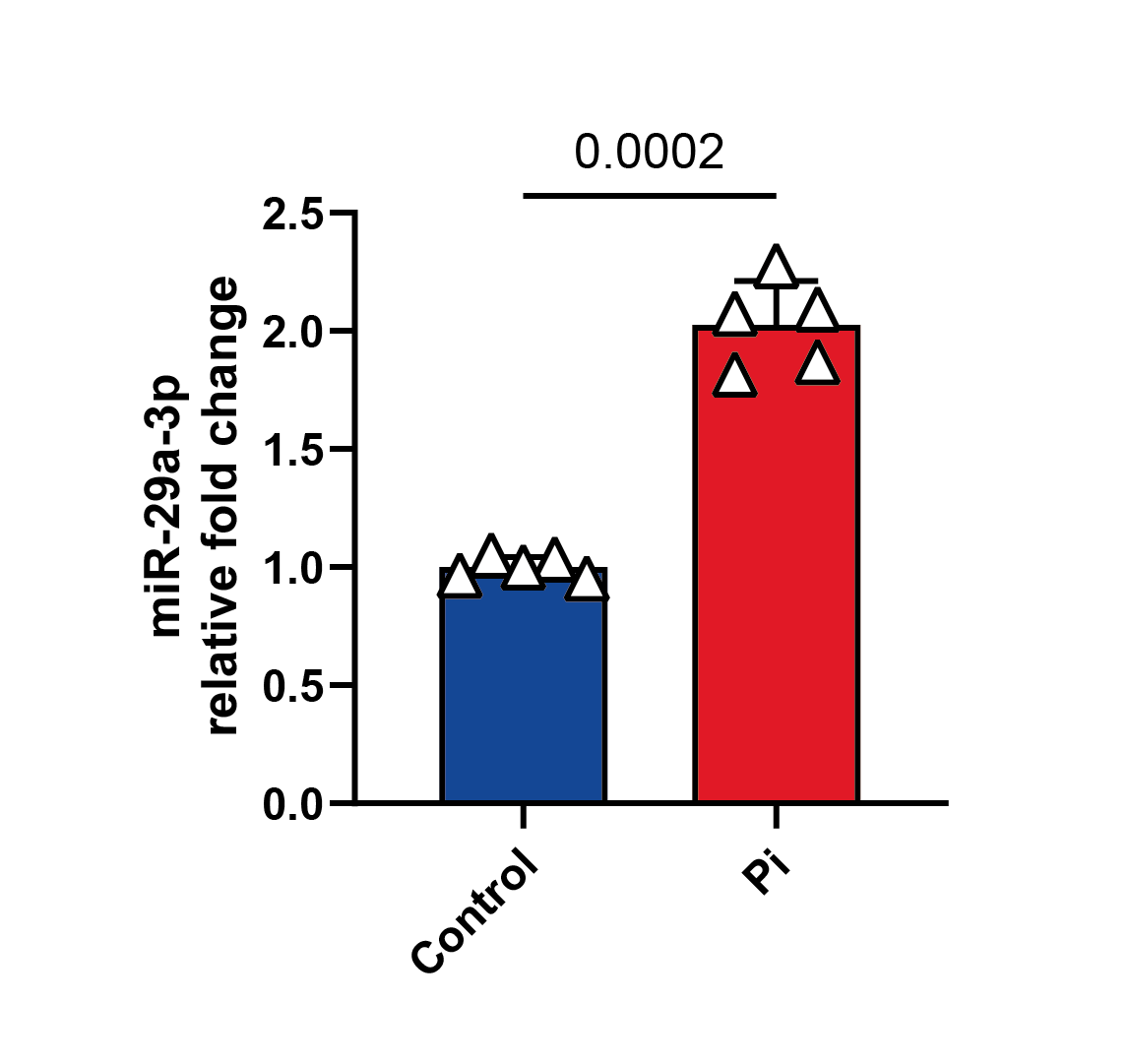

Supplement: Supp Fig 1.png [file IRNF_A_2489712_SM6942.png]
